# Supplementary material for: From sequence to enzyme mechanism using multi-label machine learning
Source: BMC Bioinformatics. 2014 May 19;15:150. doi: 10.1186/1471-2105-15-150 (PMC4229970; doi:10.1186/1471-2105-15-150)
Supplement: Additional file 2 — Java code of ml2db. Additional file ml2db_code.tar.gz contains the Java source code to run the multi-label machine learning experiments and save the results to database. The code’s Javadoc is included. [file 1471-2105-15-150-S2.zip › additional file 2/ml2db/ml2db/doc/mulan/classifier/neural/model/BasicNeuralNet.html]

BasicNeuralNet


JavaScript is disabled on your browser.


- Overview
- Package
- Class
- Use
- Tree
- Deprecated
- Index
- Help

- Prev Class
- Next Class

- Frames
- No Frames

- All Classes

- Summary:
- Nested |
- Field |
- Constr |
- Method

- Detail:
- Field |
- Constr |
- Method


mulan.classifier.neural.model

## Class BasicNeuralNet

- java.lang.Object
- - mulan.classifier.neural.model.BasicNeuralNet

- All Implemented Interfaces:
  :   java.io.Serializable, NeuralNet

  ---

    

  ```
  public class BasicNeuralNet
  extends java.lang.Object
  implements NeuralNet, java.io.Serializable
  ```

  Implementation of basic neural network. The network consists of one input layer,
  zero or more hidden layers and one output layer. Each layer contains 1 or more
  `Neuron` units. The input layer is used just to store and forward input
  pattern of the network to first hidden layer for processing.
  Input layer do not process input pattern. Neurons of input layer have one input weight
  equal to 1, bias weight equal to 0 and use linear activation function.

  Version:
  :   2012.02.27

  Author:
  :   Jozef Vilcek

  See Also:
  :   Serialized Form

- - ### Constructor Summary

    Constructors

    Constructor and Description

    `BasicNeuralNet(int[] netTopology,
    double biasInput,
    java.lang.Class<? extends ActivationFunction> activationFunction,
    java.util.Random random)`

    Creates a new `BasicNeuralNet` instance.
  - ### Method Summary

    Methods

    | Modifier and Type | Method and Description |
    | --- | --- |
    | `double[]` | `feedForward(double[] inputPattern)` Propagates the input pattern through the network. |
    | `int` | `getLayersCount()` Returns a total number of layers of the neural network. |
    | `java.util.List<Neuron>` | `getLayerUnits(int layerIndex)` Returns units of a particular layer of the neural network. |
    | `int` | `getNetInputSize()` Gets the size/dimension of the input layer of the neural network. |
    | `int` | `getNetOutputSize()` Gets the size/dimension of the output layer of the neural network. |
    | `double[]` | `getOutput()` Returns the actual output of the neural network, which is a result of last processed input pattern. |
    | `void` | `reset()` Perform reset, re-initialization of neural network. |

    - ### Methods inherited from class java.lang.Object

      `equals, getClass, hashCode, notify, notifyAll, toString, wait, wait, wait`

- - ### Constructor Detail


    - #### BasicNeuralNet

      ```
      public BasicNeuralNet(int[] netTopology,
                    double biasInput,
                    java.lang.Class<? extends ActivationFunction> activationFunction,
                    java.util.Random random)
      ```

      Creates a new `BasicNeuralNet` instance.

      Parameters:
      :   `netTopology` - defines a topology of the network. The array length corresponds
          to number of network layers. The values of the array corresponds to number
          of neurons in each particular layer.
      :   `biasInput` - the bias input value for neurons of the neural network.
      :   `activationFunction` - the type of activation function to be used by network elements
      :   `random` - the pseudo-random generator instance to be used for computations involving randomness.
          This parameter can be null. In this case, new random instance with default seed will be constructed where needed.

      Throws:
      :   `java.lang.IllegalArgumentException` - if network topology is incorrect of activation function class is null.
  - ### Method Detail


    - #### getLayerUnits

      ```
      public java.util.List<Neuron> getLayerUnits(int layerIndex)
      ```

      **Description copied from interface: `NeuralNet`**

      Returns units of a particular layer of the neural network.
      The valid indexes for layers are from 0 to N-1, where N is total number of layers

      **Specified by:**
      :   `getLayerUnits` in interface `NeuralNet`

      Returns:
      :   returns an unmodifiable list of units of the particular layer

      See Also:
      :   `Collections.unmodifiableList(List)`


    - #### getLayersCount

      ```
      public int getLayersCount()
      ```

      **Description copied from interface: `NeuralNet`**

      Returns a total number of layers of the neural network.

      **Specified by:**
      :   `getLayersCount` in interface `NeuralNet`

      Returns:
      :   the number of layers in the neural network


    - #### feedForward

      ```
      public double[] feedForward(double[] inputPattern)
      ```

      **Description copied from interface: `NeuralNet`**

      Propagates the input pattern through the network.

      **Specified by:**
      :   `feedForward` in interface `NeuralNet`

      Parameters:
      :   `inputPattern` - the input pattern for the network to process

      Returns:
      :   the output of the network


    - #### getOutput

      ```
      public double[] getOutput()
      ```

      **Description copied from interface: `NeuralNet`**

      Returns the actual output of the neural network,
      which is a result of last processed input pattern.

      **Specified by:**
      :   `getOutput` in interface `NeuralNet`

      Returns:
      :   the output of the network.
          Returns null if network is reset or no input pattern was processed


    - #### reset

      ```
      public void reset()
      ```

      **Description copied from interface: `NeuralNet`**

      Perform reset, re-initialization of neural network.
      All learned knowledge stored in the network will be lost.

      **Specified by:**
      :   `reset` in interface `NeuralNet`


    - #### getNetInputSize

      ```
      public int getNetInputSize()
      ```

      **Description copied from interface: `NeuralNet`**

      Gets the size/dimension of the input layer of the neural network.
      This is the size of input pattern the neural network can process.

      **Specified by:**
      :   `getNetInputSize` in interface `NeuralNet`

      Returns:
      :   the network input size


    - #### getNetOutputSize

      ```
      public int getNetOutputSize()
      ```

      **Description copied from interface: `NeuralNet`**

      Gets the size/dimension of the output layer of the neural network.
      This is the size of output pattern the neural network produces.

      **Specified by:**
      :   `getNetOutputSize` in interface `NeuralNet`

      Returns:
      :   the network output size


- Overview
- Package
- Class
- Use
- Tree
- Deprecated
- Index
- Help

- Prev Class
- Next Class

- Frames
- No Frames

- All Classes

- Summary:
- Nested |
- Field |
- Constr |
- Method

- Detail:
- Field |
- Constr |
- Method
